# Factors influencing the learning transfer of nursing students in a non-face-to-face educational environment during the COVID-19 pandemic in Korea: a cross-sectional study using structural equation modeling

**DOI:** 10.3352/jeehp.2023.20.14

**Published:** 2023-04-27

**Authors:** Geun Myun Kim, Yunsoo Kim, Seong Kwang Kim

**Affiliations:** 1 Department of Nursing, Gangneung Wonju National University, Wonju, Korea; 2 Department of Nursing, Catholic Kwandong University, Gangneung, Korea; Hallym University, Korea

**Keywords:** COVID-19, Transfer, Nursing students, Personal satisfaction, Republic of Korea, Latent class analysis

## Abstract

**Purpose:**

The aim of this study was to identify factors influencing the learning transfer of nursing students in a non-face-to-face educational environment through structural equation modeling and suggest ways to improve the transfer of learning.

**Methods:**

In this cross-sectional study, data were collected via online surveys from February 9 to March 1, 2022, from 218 nursing students in Korea. Learning transfer, learning immersion, learning satisfaction, learning efficacy, self-directed learning ability and information technology utilization ability were analyzed using IBM SPSS for Windows ver. 22.0 and AMOS ver. 22.0.

**Results:**

The assessment of structural equation modeling showed adequate model fit, with normed *χ*^2^=1.74 (P<0.024), goodness-of-fit index=0.97, adjusted goodness-of-fit index=0.93, comparative fit index=0.98, root mean square residual=0.02, Tucker-Lewis index=0.97, normed fit index=0.96, and root mean square error of approximation=0.06. In a hypothetical model analysis, 9 out of 11 pathways of the hypothetical structural model for learning transfer in nursing students were statistically significant. Learning self-efficacy and learning immersion of nursing students directly affected learning transfer, and subjective information technology utilization ability, self-directed learning ability, and learning satisfaction were variables with indirect effects. The explanatory power of immersion, satisfaction, and self-efficacy for learning transfer was 44.4%.

**Conclusion:**

The assessment of structural equation modeling indicated an acceptable fit. It is necessary to improve the transfer of learning through the development of a self-directed program for learning ability improvement, including the use of information technology in nursing students’ learning environment in non-face-to-face conditions.

## Graphical abstract

[Fig f3-jeehp-20-14]

## Introduction

### Background/rationale

Even after the end of the coronavirus disease 2019 (COVID-19) pandemic, non-face-to-face classes are expected to be used selectively in all educational environments in the future. In addition, as the demand for digital changes, including artificial intelligence, the internet of things, augmented reality, and virtual reality, is incorporated into the school environment, strategies for improving the quality of education are needed.

Nursing students complete a curriculum consisting of theoretical education, on-campus practice, and various clinical practices. Therefore, to effectively apply the knowledge and experience learned in theory and practice in nursing school to the clinic, it is essential to have the ability to transfer learning in order to effectively apply the acquired knowledge, skills, and behaviors to work on an ongoing basis [1]. Ford and Weissbein [2] defined learning transfer as the generalization and maintenance of knowledge and skills acquired in education and training situations for a certain period in practical situations. Keller explained the intrinsic and extrinsic aspects of the abstract concept of motivation through the attention, relevance, confidence, and satisfaction (ARCS) model and stated that intrinsic and extrinsic motivation can be enhanced by these 4 factors [3]. In other words, for learning to take place, first of all, attention must be paid, teaching contents and methods must be relevant to learners, learners must be able to have confidence in being able to succeed, and learners must be able to feel a sense of accomplishment. In a previous study, the 4 main factors mentioned in the ARCS theory by Keller [4] were reported to have a significant effect on learning immersion [5].

According to the learning transfer model of Ayres [6], the result of education is learning transfer, and factors that facilitate this process include characteristics such as the degree of the individual’s relevant knowledge, skills, abilities, and individual experience before education and the relevance of the educational program. It was shown that personal and environmental factors also affect learners’ learning satisfaction, which is a factor linked to learning motivation [6]. These factors are related to the design factors of the educational program itself, meaning that learning transfer should be effective. In particular, the ability to use information technology (IT) in a non-face-to-face class situation can be considered a personal characteristic.

Most prior studies on learning transfer have been conducted among entrepreneurs [7]. Therefore, in this study, based on the theories of Keller [4] and Ayres [6] related to learning transfer, a model explaining the relationship between various factors affecting learning transfer in nursing students was constructed and verified.

### Objectives

The aim of the study was to identify the factors influencing the learning transfer of nursing students in a non-face-to-face educational environment during the COVID-19 pandemic through structural equation modeling. Specifically, a structural equation model was constructed and the model fitness for paths affecting learning transfer was tested.

## Methods

### Ethics statement

This study received approval from the Ethics Committee (Institutional Review Board) of Gangneung Wonju National University (2021-33-1). Informed consent was obtained from the participants during the first part of the online survey.

### Study design

**Fig. 1.**
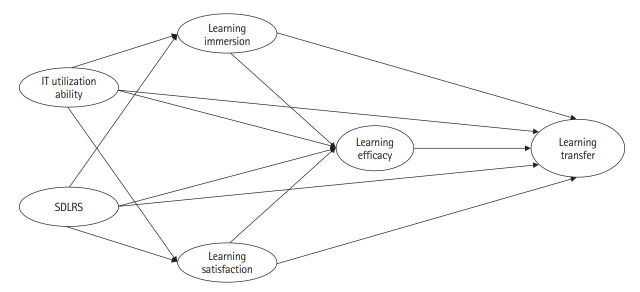
Hypothetical framework. IT, information technology; SDLRS, Self-Directed Learning Readiness Scale.

This was a cross-sectional study. A hypothetical model was constructed based on the learning transfer model of Keller [4] and Ayres [6] and related literature as follows (Fig. 1).

### Setting

Data were collected using a Google online survey from February 9 to March 1, 2022. The survey was distributed to nursing students at Gangneung Wonju National University.

### Participants

The targets of this study were 218 nursing students (83.9% female) aged over 20 years (mean±standard deviation [SD]=22.25±2.56 years). The inclusion criterion was all nursing students who were registered at the university. There were no exclusion criteria.

### Variables

There were 6 variables in this study: learning transfer, learning immersion, learning satisfaction, learning self-efficacy, self-directed learning ability, and IT utilization ability. Self-directed learning ability and IT utilization ability were exogenous variables. Learning immersion, learning satisfaction, and learning self-efficacy were endogenous variables to make a hypothetical model of factors that affect learning transfer. Self-directed learning ability refers to the type of learning in which the entire process related to learning is selected and decided according to one’s own will, and IT utilization ability refers to the ability to use information and communication devices. Learning immersion means a state of psychological concentration where one is deeply immersed in learning utilization, learning satisfaction denotes satisfaction with the learning process and results, and learning self-efficacy denotes personal belief in whether the relevant content is learned.

### Data sources/measurement

In this study, a questionnaire containing 81 items in 6 categories was used: learning transfer (4 items, 5-point Likert scale) developed by Lee [8] based on the research of Rouiller and Goldstein [9]; learning immersion (29 items, 5-point Likert scale) developed by Kim et al. [10]; learning satisfaction (15 items, 5-point Likert scale) developed by Bae et al. [7]; learning self-efficacy (10 items, 7-point Likert scale) developed by Ayres [6] and translated into Korean by Park and Kweon [11]; self-directed learning ability (22 items, 5-point Likert scale) developed by Guglielmino [12] and modified by Kim and Lee [13]; and IT utilization ability (single item, 5-point Likert scale). The 5-point Likert scale extended from “extremely disagree” (1) to “extremely agree” (5), and the 7-point Likert scale ranged from “extremely disagree” (1) to “extremely agree” (7). The Cronbach α values of all measurement tools were more than 0.7, indicating reliability.

### Bias

No bias was found in selecting participants.

### Study size

The sample size met the criterion according to which more than 200 samples are required to verify a structural equation model [14]. Data were collected from 218 students, and all 218 responses were used in the final analysis.

### Statistical methods

The collected data were analyzed using IBM SPSS for Windows ver. 22.0 (IBM Corp.) and AMOS ver. 22.0 (IBM Corp.). The variables related to participants’ general characteristics were analyzed in terms of frequency, percentage, mean, and SD as descriptive statistics. The multivariate normality of the sample was verified by mean values, SD, skewness, and kurtosis. The model fit was tested using the *χ*^2^ test (chi-square value, CMIN), normed *χ*^2^ test (CMIN/degrees of freedom), goodness-of-fit index (GFI), adjusted goodness-of-fit index (AGFI), comparative fit index (CFI), a non-normed fit index (Tucker-Lewis index, TLI), normed fit index (NFI), standardized root mean square residual (SRMR), and root mean square error of approximation (RMSEA). The significance of the estimated coefficient for each path in the hypothetical model was analyzed through the critical ratio and P-value (P<0.05). The bootstrapping method was used to assess the statistical significance of the direct, indirect, and total effects of the hypothetical model.

## Results

### Participants

All of the 218 targeted nursing students participated in this survey. Their average age was 22.25 years. Most (83.9%) were women, 44.5% were in the fourth year of their program, and 72.9% were living with their parents. The average score for self-perceived IT utilization ability was 3.79 points, that for academic achievement was 3.24 points, and that for major satisfaction was 3.79 points. The most common form of non-face-to-face classes experienced at university was real-time online classes and video-recorded lectures (82.1%). Furthermore, 55.0% of participants responded that they had experience in clinical practice, 82.6% stated that they had experience in laboratory practice, and 54.1% reported that they had experience in simulation practice (Table 1).

### Main results

#### Descriptive statistics and confirmatory factor analysis of the measured variables

The descriptive statistics of the 6 measured variables are presented in Table 2 (Dataset 1). Confirmatory factor analysis was performed to evaluate the degree of validity of the factors. Three items with a standard factor loading (λ) <0.50 were excluded. The standard factor loading of the other variables was above the baseline value (λ>0.5), the construct validity was above 0.80, and the average was above 0.50, indicating no issue with convergent validity.

We further examined the correlations between the correlation matrix and the average to verify discriminant validity. This analysis demonstrated that the values of the multiple correlation coefficients were small enough to ensure the factorial discriminant validity, with values ranging between 0.04 and 0.80 (i.e., with absolute values less than 0.85).

#### Goodness-of-fit testing of the model

Prior to hypothetical model verification, confirmatory factor analysis demonstrated that the subjective IT utilization ability, self-directed learning ability, learning satisfaction, learning immersion, and learning self-efficacy of nursing students’ learning transfer were relatively good, and the hypothetical model was verified without model modification. The fit indices of the model in this study were as follows: *χ*^2^=33.07, normed *χ*^2^=1.74 (P=0.024), GFI=0.97, AGFI=0.93, CFI=0.98, RMR=0.02, TLI=0.97, NFI=0.96, and RMSEA=0.06 (Table 3).

#### Analysis of the hypothetical model

**Fig. 2.**
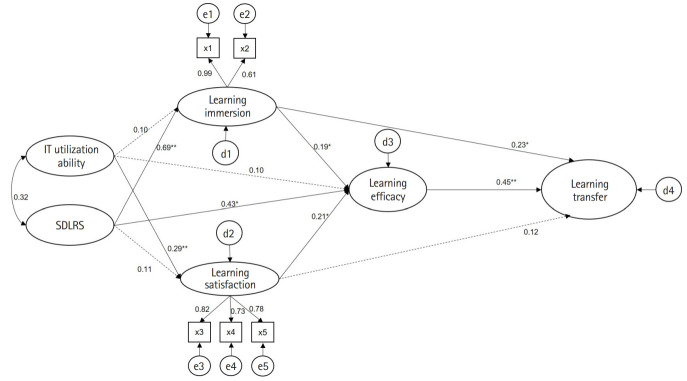
Effect analysis in the structural equation model. IT, information technology; SDLRS, Self-Directed Learning Readiness Scale; X1, cognitive immersion; X2, affective immersion; X3, systematic satisfaction; X4, reactive satisfaction; X5, affirmative satisfaction. *P<0.05. **P<0.01.

The hypothetical model analysis showed that 9 out of the 11 pathways of the hypothetical structural model for learning transfer in nursing students were statistically significant (Fig. 2). For learning immersion, subjective IT utilization ability (β=0.099, P=0.047) and initiative (β=0.687, P<0.001) appeared as statistically significant paths. The explanatory power of IT utilization ability and self-directed learning ability for learning immersion was 52.5%. For learning satisfaction, subjective IT utilization ability (β=0.291, P<0.001) appeared as a statistically significant path, and the explanatory power of variables for learning satisfaction was 11.8%. For learning self-efficacy, initiative (β=0.429, P<0.001), immersion (β=0.187, P=0.016), and satisfaction (β=0.211, P<0.001) were identified as significant pathways, and the explanatory power of initiative, learning flow, and learning satisfaction for learning satisfaction was 49.3%. For learning transfer, learning immersion (β=0.232, P<0.001), learning satisfaction (β=0.121, P=0.048), and learning self-efficacy (β=0.454, P<0.001) were found to be significant pathways. The explanatory power of immersion, satisfaction, and self-efficacy for learning transfer was 44.4% (Tables 4, 5 and Fig. 2).

## Discussion

### Key results

Nursing students’ learning self-efficacy and learning flow directly affected learning transfer, and subjective IT utilization ability, self-directed learning ability, and learning satisfaction were variables with indirect effects. The explanatory power of variables influencing the learning transfer of nursing students was 44.4%.

### Interpretation

Self-directed learning ability did not affect learning transfer directly; instead, it seemed to affect learning transfer through learning self-efficacy. Learning self-efficacy was found to be the most influential factor in learning transfer among nursing students. The factor that had the most significant influence on learning self-efficacy was self-directed learning ability. In an educational environment that has suddenly changed to non-face-to-face due to COVID-19, students learn independently and autonomously without the support of others; however, the ability and behavioral elements that individuals have for leading learning activities are derived from their experiences with face-to-face education. This behavioral change in learning activities among students has a greater influence than in the context of distance learning. It is not easy to immediately ascertain whether a student has understood the learning content conveyed through certain content. When learning occurs in a situation where teachers and students are not face-to-face, students’ ability and behavior in self-directed learning activities have a more significant impact than in face-to-face education situations.

The next factors influencing the learning transfer of nursing students were learning immersion and learning satisfaction. Satisfaction with learning can enhance the knowledge and skills that students need to learn by themselves by increasing their confidence in learning or self-efficacy.

Finally, nursing students’ IT utilization ability was found to have an indirect effect on their learning transfer. In the environment that suddenly changed after the start of the COVID-19 pandemic, nursing education was converted to non-face-to-face education, for both theoretical and practical education. IT utilization ability is essential in the remote education environment in the era of COVID-19.

### Comparison with previous studies

In the non-face-to-face educational environment implemented during the COVID-19 pandemic, interaction between students and professors and between students is difficult; thus, immersion in learning and satisfaction with learning may decrease. Studies have also shown benefits of social interactions in the lecture for students’ learning [15]. A sense of belonging is often fostered through academic social interactions, and if students do not feel like they belong, they may be less driven to complete their academic work [16]. Students have often experienced loneliness during the pandemic, and the lack of face-to-face social interactions during the pandemic is not only connected to feelings of isolation, but might also be a serious cause of stress for students [17].

### Limitations/generalizability

This study collected data from nursing students in 2 regions of Korea, and the results might not be fully generalizable to all nursing students and all college students. In addition, since the path from learning transfer to learning performance was not analyzed in this study, it cannot be asserted that all of the students achieved learning transfer to learning performance.

### Implications

For learning activities in a non-face-to-face environment, such as non-face-to-face classes, expectations are rising for the content presented and the ability to use IT in the learning environment to present the learning content. In addition, an active information processing process is required, such as constructing new knowledge based on individual abilities. Therefore, it is necessary to improve the transfer of learning through the development of a self-directed program for learning ability improvement, including the use of IT in the learning environment of nursing students in a non-face-to-face situation.

### Conclusion

In this structural equation model, subjective IT utilization ability, initiative, immersion, learning satisfaction, and learning self-efficacy had an explanatory power of 44.4% (i.e., middle level) for learning transfer among nursing students. Because the personal factors and learning motivation variables selected in the hypothetical model of this study were appropriately selected to explain nursing students’ learning transfer, it is judged that the variables are appropriate. Therefore, the variables presented in this study must be considered as influencing factors when developing a program to improve the learning transfer of nursing students in the future.

## Figures and Tables

**Figure f3-jeehp-20-14:**
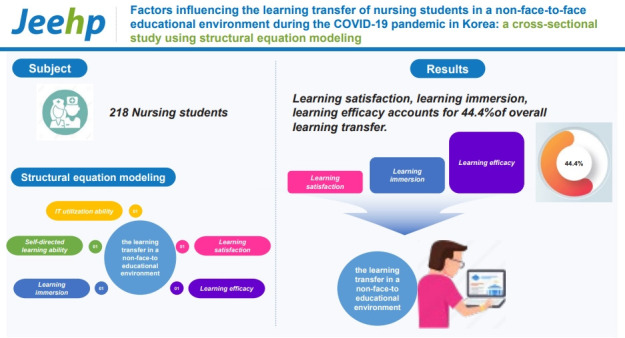


**Table 1.** General characteristics of the participants (N=218)

Characteristic	Category	Value
Age (yr)		22.25±2.56
Gender	Male	35 (16.1)
	Female	183 (83.9)
Year	1st	8 (3.7)
	2nd	30 (13.8)
	3rd	83 (38.1)
	4th	97 (44.5)
Residence	With parents	159 (72.9)
	Living alone	52 (23.9)
	Dormitory	6 (2.8)
	Other	1 (0.5)
Subjective academic achievement		3.24±1.02
Major satisfaction		3.79±0.82
Class type	Online live class	28 (12.8)
	Video lecture	10 (4.8)
	Both	179 (82.1)
	Other	1 (0.5)
Experience of clinical practice	Yes	120 (55.0)
	No	98 (45.0)
Lab practice	Yes	180 (82.6)
	No	38 (17.4)
Simulation practice	Yes	118 (54.1)
	No	100 (45.9)

Values are presented as mean±standard deviation or number (%).

**Table 2.** Descriptive statistics of the measured variables (N=218)

Variable	Mean±SD	Measurement range	Skewness	Kurtosis
IT utilization ability	3.79±0.86	1.00–5.00	-0.370	0.027
Self-directed learning ability	3.67±0.42	2.45–4.86	0.098	0.055
Learning satisfaction				
Systematicity	3.44±0.69	1.20–5.00	-0.149	0.441
Reactivity	3.58±0.74	1.25–5.00	-0.125	0.167
Certainty	4.02±0.58	2.00–5.00	-0.429	0.514
Learning immersion				
Cognitive immersion	3.39±0.61	1.13–5.00	-0.350	0.779
Affective immersion	2.73±0.69	1.00–4.43	-0.162	0.048
Learning efficacy	5.66±0.80	2.00–7.00	-0.994	1.951
Learning transfer	3.84±0.57	1.00–5.00	0-.782	3.225

SD, standard deviation.

**Table 3.** Model fit (N=218)

Model	χ^2^	χ^2^/df	P-value	GFI	AGFI	CFI	RMR	TLI	NFI	RMSEA
Modified model	33.07	1.74	0.024	0.97	0.93	0.98	0.02	0.97	0.96	0.06

df, degrees of freedom; GFI, goodness-of-fit index; AGFI, adjusted goodness-of-fit index; CFI, comparative fit index; RMR, root mean square residual; TLI, Tucker-Lewis index; NFI, normed fit index; RMSEA, root mean square error of approximation.

**Table 4.** Summary of structural model (N=218)

Endogenous variable	Category	Estimate	Standardized estimate	CR	SMC
Learning immersion	IT utilization ability	0.099 (<0.047)	0.035	1.986	0.525
	Self-directed learning ability	0.687 (<0.001)	0.072	13.744	
Learning satisfaction	IT utilization ability	0.291 (<0.001)	0.041	3.791	0.118
	Self-directed learning ability	0.113	0.081	1.500	
Learning efficacy	IT utilization ability	0.101	0.051	1.847	0.493
	Self-directed learning ability	0.429 (<0.001)	0.141	5.779	
	Learning immersion	0.187 (0.016)	0.105	2.416	
	Learning satisfaction	0.211 (<0.001)	0.102	3.550	
Learning transfer	Learning immersion	0.232 (<0.001)	0.064	3.459	0.444
	Learning satisfaction	0.121 (0.048)	0.078	1.978	
	Learning efficacy	0.454 (<0.001)	0.049	6.704	

CR, critical ratio; SMC, squared multiple correlation; IT, information technology.

**Table 5.** Direct, indirect, and total effects in the model (N=218)

Endogenous variable	Category	Direct effect		Indirect effect		Total effect	
		B	P-value	B	P-value	B	P-value
Learning immersion	IT utilization ability	0.099	0.051			0.099	0.051
	Self-directed learning ability	0.687	0.009			0.687	0.009
Learning satisfaction	IT utilization ability	0.291	0.007			0.291	0.007
	Self-directed learning ability	0.113	0.315			0.113	0.315
Learning efficacy	IT utilization ability	0.101	0.182	0.080	0.013	0.181	0.017
	Self-directed learning ability	0.429	0.012	0.152	0.007	0.581	0.016
	Learning immersion	0.187	0.010			0.187	0.010
	Learning satisfaction	0.211	0.012			0.211	0.012
Learning transfer	IT utilization ability			0.140	0.010	0.140	0.010
	Self-directed learning ability			0.437	0.015	0.437	0.013
	Learning immersion	0.232	0.037	0.085	0.013	0.317	0.010
	Learning satisfaction	0.121	0.178	0.096	0.010	0.217	0.015
	Learning efficacy	0.454	0.008			0.454	0.008

IT, information technology.

## Footnotes

**Data availability**
Data files are available from Harvard Dataverse: https://doi.org/10.7910/DVN/3JDN9S Dataset 1. Raw data of responses from South Korean nursing students in 2021.

## Supplementary materials

Supplementary files are available from Harvard Dataverse: https://doi.org/10.7910/DVN/3JDN9S

Supplement 1. Audio recording of the abstract.

## References

[1] Noe R (2005). Employee training and development.

[2] Ford JK, Weissbein DA (1997). Transfer of training: an updated review and analysis. Perform Improv Q.

[3] Han KS, Park YJ, Kim KM, Oh YJ, Jin JH, Kang HC (2008). Communication style, self efficacy, emotional regulation, and ways of coping among nursing students. J Korean Acad Psychiatr Ment Health Nurs.

[4] Keller JM (1987). Development and use of the ARCS model of instructional design. J Instr Dev.

[5] Cai X, Li Z, Zhang J, Peng M, Yang S, Tian X, Yang Q, Yan F (2022). Effects of ARCS model-based motivational teaching strategies in community nursing: a mixed-methods intervention study. Nurse Educ Today.

[6] Ayres HW (2005). Factors related to motivation to learn and motivation to transfer learning in a nursing population [dissertation].

[7] Bae EK, Jang MY, Kim DY (2009). Learning transfer and its determinant factors in different types of organization: company, school, hospital in Incheon. Andragogy Today Int J Adult Contin Educ.

[8] Lee DH (1996). A model testing study on the learning and transfer of training in organizations. Korean J Ind Organ Psychol.

[9] Rouiller JZ, Goldstein IL (1993). The relationship between organizational transfer climate and positive transfer of training. Hum Resour Dev Q.

[10] Kim A, Tack H, Lee CH (2010). The development and validation of a learning flow scale for adults. Korean J Educ Psychol.

[11] Park SY, Kweon YR (2012). The effect of using standardized patients in psychiatric nursing practical training for nursing college students. J Korean Acad Psychiatr Ment Health Nurs.

[12] Guglielmino LM (1978). Development of the Self-Directed Learning Readiness Scale. Diss Abstr Int Sect A Humanit Soc Sci.

[13] Kim K, Lee G (2020). Analysis of structural relations among self-directed learning, learning flow, academic self-efficacy, career decision self-efficacy, and key competencies of college students. J Yeolin Educ.

[14] Hoelter JW (1983). The analysis of covariance structures: goodness-of-fit indices. Sociol Methods Res.

[15] Hurst B, Wallace RR, Nixon SB (2013). The impact of social interaction on student learning. Read Horiz J Lit Lang Arts.

[16] Yeager D, Walton G, Cohen GL (2013). Addressing achievement gaps with psychological interventions. Phi Delta Kappan.

[17] Labrague LJ, De Los Santos JA, Falguera CC (2021). Social and emotional loneliness among college students during the COVID-19 pandemic: the predictive role of coping behaviors, social support, and personal resilience. Perspect Psychiatr Care.

